# CTGF Increases IL-6 Expression in Human Synovial Fibroblasts through Integrin-Dependent Signaling Pathway

**DOI:** 10.1371/journal.pone.0051097

**Published:** 2012-12-05

**Authors:** Shan-Chi Liu, Chin-Jung Hsu, Hsien-Te Chen, Hsi-Kai Tsou, Show-Mei Chuang, Chih-Hsin Tang

**Affiliations:** 1 Institute of Biomedical Sciences, National Chung Hsing University, Taichung, Taiwan; 2 School of Chinese Medicine, College of Chinese Medicine, China Medical University, Taichung, Taiwan; 3 Department of Orthopedic Surgery, China Medical University Hospital, Taichung, Taiwan; 4 Department of Materials Science and Engineering, Feng Chia University, Taichung, Taiwan; 5 Department of Neurosurgery, Taichung Veterans General Hospital, Taichung, Taiwan; 6 Center for General Education, Jen-Teh Junior College of Medicine, Nursing and Management, Miaoli County, Taiwan; 7 Department of Pharmacology, School of Medicine, China Medical University, Taichung, Taiwan; 8 Graduate Institute of Basic Medical Science, China Medical University, Taichung, Taiwan; National Center for Scientific Research Demokritos, Greece

## Abstract

**Background:**

Connective tissue growth factor (CTGF; also known as CCN2) is an inflammatory mediator, and shows elevated levels in regions of severe injury and inflammatory diseases. CTGF is abundantly expressed in osteoarthritis (OA). However, the relationship between CTGF and IL-6 in OA synovial fibroblasts (OASFs) is mostly unknown.

**Methodology/Principal Findings:**

OASFs showed significant expression of CTGF, and expression was higher than in normal SFs. OASFs stimulation with CTGF induced concentration-dependent increases in IL-6 expression. CTGF mediated IL-6 production was attenuated by αvβ5 integrin neutralized antibody and apoptosis signal-regulating kinase 1 (ASK1) shRNA. Pretreatment with p38 inhibitor (SB203580), JNK inhibitor (SP600125), AP-1 inhibitors (Curcumin and Tanshinone IIA), and NF-κB inhibitors (PDTC and TPCK) also inhibited the potentiating action of CTGF. CTGF-mediated increase of NF-κB and AP-1 luciferase activity was inhibited by SB203580 and SP600125 or ASK1 shRNA or p38 and JNK mutant.

**Conclusions/Significance:**

Our results suggest that CTGF increased IL-6 production in OASFs via the αvβ5 integrin, ASK1, p38/JNK, and AP-1/NF-κB signaling pathways.

## Introduction

Osteoarthritis (OA) is the most common form of arthritis, and is the single most important cause of disability in older adults [1]. The exact etiology of OA is not well understood [2]. In response to the proinflammatory cytokines produced by macrophages, such as interleukin-1β and tumor necrosis factor-α, osteoarthritis synovial fibroblasts (OASFs) produce chemokines that promote inflammation, neovascularization, and cartilage degradation via activation of matrix-degrading enzymes, such as matrix metalloproteinases [3]. Diagnosis of the disease and the progression of joint damage are mainly based on evaluation of clinical and radiological findings. Molecular markers can serve as promising indicators for OA evaluation because they can provide more direct information about the local inflammation, the alterations in joint tissues and related bone and cartilage turnover [4].

IL-6 is a multifunctional cytokine that plays a central role in both innate and acquired immune responses. It is the predominant mediator of the acute phase response, an innate immune mechanism which is triggered by infection and inflammation [5], [6]. In addition to these roles in pathogen specific inflammation and immunity, IL-6 levels are elevated in chronic inflammatory conditions, such as OA [7], [8]. It has been reported the concentration of IL-6 in OA synovial fluid is positively correlated with the total leukocyte count [9]. A clinical trial in patients with OA showed that high baseline levels of IL-6 were associated with an increased risk of cartilage loss. Several consensus sequences, including those for NF-κB, CREB, NF-IL-6, and AP-1 in the 5′ promoter region of the IL-6 gene, have been identified as regulatory sequences that induce IL-6 in response to various stimuli [10], [11].

Connective tissue growth factor (CTGF; also call CCN2) is a member of the CCN family, which is a group of secreted multifunctional proteins that contain high levels of cysteine [12]. It has been shown that CTGF is associated with several biological functions such as fibrosis, tumorgenesis, and angiogenesis even to OA [12], [13]. CTGF mRNA has been found to be up-regulated adjacent to areas of cartilage surface damage, and present in chondro-osteophytes [14]. In animal model, CTGF overexpression in mouse knee joints induces fibrosis and cartilage damage [15]. The cartilage damage that was found could be either a direct effect of CTGF overexpression or a result of factors excreted by the CTGF-induced fibrotic synovial tissue [15]. Therefore, CTGF is a likely candidate to contribute the pathogenesis of OA.

Previous studies have shown that CTGF promotes inflammatory response [16], [17]. Although a role for CTGF in IL-6 induction has been implied for some cell types, the signaling pathway for CTGF in IL-6 production in synovial fibroblasts has not been extensively studied. In the present study, we explored the intracellular signaling pathway involved in CTGF-induced IL-6 production in human synovial fibroblast cells. The results showed that CTGF activates αvβ5 integrin, apoptosis signal-regulating kinase 1 (ASK1), p38/JNK, and AP-1/NF-κB pathways, leading to up-regulation of IL-6 expression.

## Materials and Methods

### Materials

Anti-mouse and anti-rabbit IgG-conjugated horseradish peroxidase, rabbit polyclonal antibodies specific for β-actin, ASK1, p-JNK, JNK, p-p38, p38, p-c-Jun, c-Jun, p-IKK, IKK, p-IκB, IκB, p-p65, p65 and the small interfering RNAs (siRNAs) against ASK1, c-Jun, and a control for experiments using targeted siRNA transfection (each consists of a scrambled sequence that does not lead to specific degradation of any known cellular mRNA) were purchased from Santa Cruz Biotechnology (Santa Cruz, CA). Rabbit polyclonal antibodies specific for ASK1 phosphorylated at Thr^845^ and Ser^967^ were purchased from Cell Signaling and Neuroscience (Danvers, MA). Mouse monoclonal antibodies specific for αvβ3, αvβ5, α5β1, and α6β1 integrin were purchased from Millipore Biotechnology (Billerica, MA). The recombinant human CTGF and CTGF and IL-6 ELISA kit were purchased from PeproTech (Rocky Hill, NJ, USA). Tanshinone IIA was purchased from BIOMOL (Butler Pike, PA). The NF-κB and AP-1 luciferase plasmids were from Stratagene (La Jolla, CA). The IKKα(KM) and IKKβ (KM) mutants were provided by Dr. H. Nakano (Juntendo University, Tokyo, Japan). The p38 dominant negative mutant was provided by Dr. J. Han (University of Texas South-western Medical Center, Dallas, TX). The JNK dominant negative mutant was provided by Dr. M. Karin (University of California, San Diego, CA, USA). The human IL-6 promoter construct pIL6-luc651(−651/+1), AP-1 site mutation (pIL6-luc651ΔAP-1), NF-κB site mutation (pIL6-luc651ΔNF-κB), and C/EBP-β site mutation (pIL6-luc651ΔC/EBP-β) were provided by Dr. O. Eickelberg (Department of Medicine II, University of Giessen, Giessen, Germany). The pSV-β-galactosidase vector and luciferase assay kit were purchased from Promega (Madison, WI). All other chemicals were purchased from Sigma-Aldrich (St. Louis, MO).

### Cell Culture

We obtained approval from the Institutional Review Board China Medical University Hospital, and subjects gave informed written consent. Human synovial fibroblasts were isolated by collagenase treatment of synovial tissue samples obtained from patients with OA during knee replacement surgeries and samples of non-arthritic synovial tissues obtained at arthroscopy after trauma/joint derangement. OASFs were isolated, cultured, and characterized as previously described [18], [19]. Experiments were performed using cells from passages 3 to 6.

### Quantitative Real-time PCR

Total RNA was extracted from synovial fibroblasts using a TRIzol kit (MDBio Inc., Taipei, Taiwan). The reverse transcription reaction was performed using 2 µg of total RNA that was reverse transcribed into cDNA using oligo (dT) primer [20], [21]. The quantitative real-time PCR (qPCR) analysis was carried out using Taqman® one-step PCR Master Mix (Applied Biosystems, Foster City, CA). cDNA templates (2 µl) were added per 25-µl reaction with sequence-specific primers and Taqman® probes. Sequences for all target gene primers and probes were purchased commercially (β-actin was used as internal control) (Applied Biosystems). The qPCR assays were carried out in triplicate on a StepOnePlus sequence detection system. The cycling conditions involved 10-min polymerase activation at 95°C, followed by 40 cycles at 95°C for 15 s and 60°C for 60 s. The threshold was set above the non-template control background and within the linear phase of the target gene amplification to calculate the cycle number at which the transcript was detected (denoted CT).

### Western Blot Analysis

Cellular lysates were prepared as described previously [22], [23]. Proteins were resolved on SDS-PAGE and transferred to Immobilon polyvinyldifluoride (PVDF) membranes. The blots were blocked with 4% BSA for 1 h at room temperature and then probed with rabbit anti-human antibodies against ASK1, p38, or JNK (1∶1000) for 1 h at room temperature. After 3 washes, the blots were subsequently incubated with donkey anti-rabbit peroxidase-conjugated secondary antibody (1∶3000) for 1 h at room temperature. The blots were visualized by enhanced chemiluminescence with Kodak X-OMAT LS film (Eastman Kodak, Rochester, NY).

### Measurements of IL-6 Production

Human synovial fibroblasts were cultured in 24-well culture plates. After reaching confluence, cells were treated with CTGF, and then incubated in a humidified incubator at 37°C for 24 h. For examination of the downstream signaling pathways involved in CTGF treatment, cells were pretreated with various inhibitors [including antibody against αvβ3, αvβ5, α5β1, and α6β1 integrin or SB203580, SP600125, U0126, PD98059, TPCK, PDTC, NF-κB inhibitor peptide, Curcumin, and Tanshinone IIA. However, these antibody or inhibitor did not affect the basal level on IL-6 expression (Fig. S1)] for 30 min before CTGF (10 ng/ml) administration. After incubation, the medium was removed and stored at −80°C until assay. IL-6 in the medium was assayed using the IL-6 enzyme immunoassay kits, according to the procedure described by the manufacturer [18], [24].

### Transfection and Reporter Gene Assay

Human synovial fibroblasts were co-transfected with 0.8 µg luciferase plasmid and 0.4 µg β-galactosidase expression vector. OASFs were grown to 80% confluence in 12 well plates and were transfected on the following day by Lipofectamine 2000 (LF2000; Invitrogen). DNA and LF2000 were premixed for 20 min and then applied to the cells. After 24 h transfection, the cells were incubated with the indicated agents. After a further 24 h incubation, the media were removed, and cells were washed once with cold PBS. To prepare lysates, 100 µl reporter lysis buffer (Promega, Madison, WI) was added to each well, and cells were scraped from dishes. The supernatant was collected after centrifugation at 13,000 rpm for 2 min. Aliquots of cell lysates (20 µl) containing equal amounts of protein (20–30 µg) were placed into wells of an opaque black 96-well microplate. An equal volume of luciferase substrate was added to all samples, and luminescence was measured in a microplate luminometer. The value of luciferase activity was normalized to transfection efficiency monitored by the co-transfected β-galactosidase expression vector.

### Synthesis of NF-κB and AP-1 Decoy Oligonucleotide

We used a phosphorothioate double-stranded decoy oligonucleotide (ODN) carrying the NF-κB-consensus sequence 5′-CCTTGAAGGGATTTCCCTCC-3′/3′-GGAACTT CCCTAAAGGGAGG-5′. The AP-1 decoy ODN sequence was 5′-TGTCTGACTCATGTC-3′/3′-ACAGACTGAGTACAG-5′. The mutated (scrambled) form 5′-TTGCCGTACCTGACTTAGCC-3′/3′-AACGGCATGGACTGAATCGG-5′ was used as a control. ODN (5 µM) was mixed with LF2000 (10 µg/ml) for 30 min at room temperature, and the mixture was added to cells in serum-free medium. After 24 h of transient transfection, the cells were used for the following experiments.

### Chromatin Immunoprecipitation Assay

Chromatin immunoprecipitation analysis was performed as described previously [24]. DNA immunoprecipitated with an anti-p65 or c-Jun Ab was purified and extracted with phenol-chloroform. The purified DNA pellet was subjected to PCR. PCR products were then resolved by 1.5% agarose gel electrophoresis and visualized with UV light. The primers 5′-CAAGACATGCCAAAGTGCTG-3′ and 5′-TTGAGACTCATGGGAAAATCC-3′ were utilized to amplify across the human IL-6 promoter region contain NF-κB binding site (−288 to −39). The primers 5′-GAACTGACCTGACTTACATA-3′ and 5′-TTGAGACTCATGGGAAAATCC-3′ were utilized to amplify across the human IL-6 promoter region contain AP-1 binding site (−312 to −39).

### Statistical Analysis

Data were expressed as means ± S.E. Statistical analysis was performed with Graphpad Prism 4. Analysis of variance (ANOVA) and unpaired two-tailed student *t* test were used to determine the significant differences between the means. *p* <0.05 was considered significant.

## Results

### CTGF Induces IL-6 Expression through αvβ5 Integrin in Human Synovial Fibroblasts

**Figure 1:**
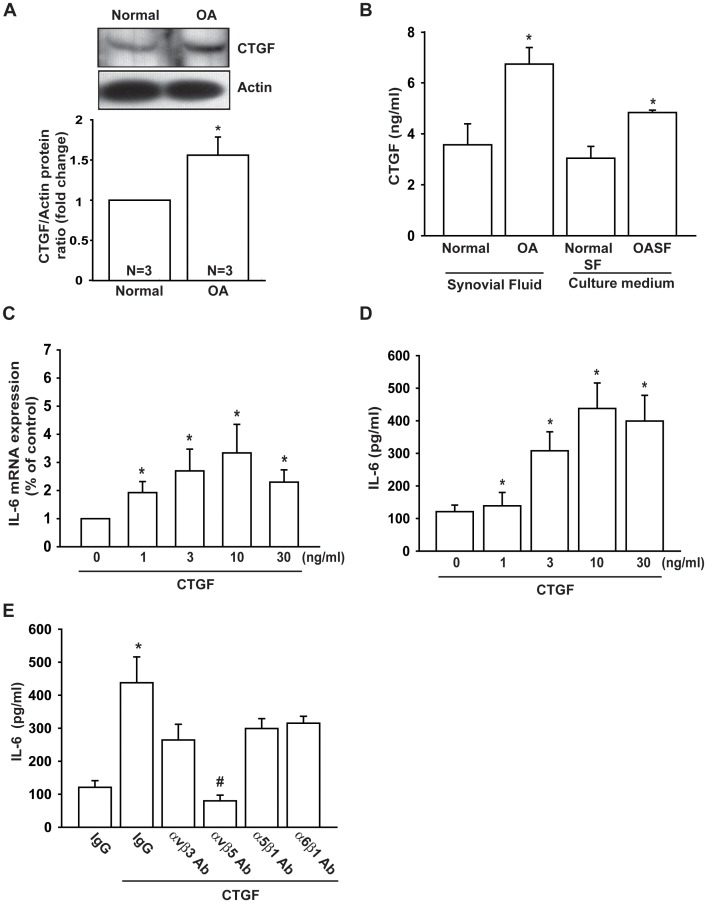
CTGF increases IL-6 expression in human synovial fibroblasts through αvβ5 integrin. (A) Total protein were extracted from normal synovial fibroblasts and OASFs, and subjected to western blotting for CTGF. (B) Human synovial fibroblasts were cultured for 48 h, and media were collected to measure CTGF. Synovial fluid was obtained from normal or osteoarthritis patients and examined with ELISA for the expression of CTGF. (C&D) OASFs were incubated with various concentrations of CTGF for 24 h. The IL-6 expression was examined by qPCR and ELISA. (E) OASFs were pretreated with αvβ3, αvβ5, α5β1, and α6β1 integrin antibody (5 µg/ml) for 30 min and then incubated with CTGF (10 ng/ml) for 24 h, the IL-6 expression was examined by ELISA. Results are expressed as the mean ± S.E. *: p<0.05 as compared with basal level. #: p<0.05 as compared with CTGF-treated group.

It have been reported that CTGF is associated with pathogenesis of OA [12], [13]. Therefore, we examined the levels of CTGF expression in samples from patients with OA and found that the expression of CTGF protein in human OASFs was significantly higher than in normal SFs (Fig. 1A). The OASFs medium showed a level of expression of CTGF that was significantly higher than that seen in the medium from normal SFs (Fig. 1B). In addition, concentrations of CTGF in synovial fluid were significantly higher in patients with OA than in controls (Fig. 1B). We applied CTGF directly to OASFs to examine the expression of IL-6, an inflammatory response gene. Treatment of OASFs with CTGF (1–30 ng/ml) for 24 h induced IL-6 mRNA and protein expression in a concentration-dependent manner (Fig. 1C&D). Previous studies have shown CTGF affects cell functions through integrin receptor signaling [25], [26]. We therefore hypothesized that integrin receptor signaling pathway may be involved in CTGF-mediated IL-6 expression in OASFs. Pretreatment of cells for 30 min with anti-αvβ5 but not anti-αvβ3, α5β1, and α6β1 monoclonal antibody (mAb) markedly reversed the CTGF-induced IL-6 production (Fig. 1E). These results indicate that CTGF increased IL-6 expression in human OASFs via αvβ5 integrin receptor.

### Involvement of ASK1 in CTGF-induced IL-6 Expression

**Figure 2:**
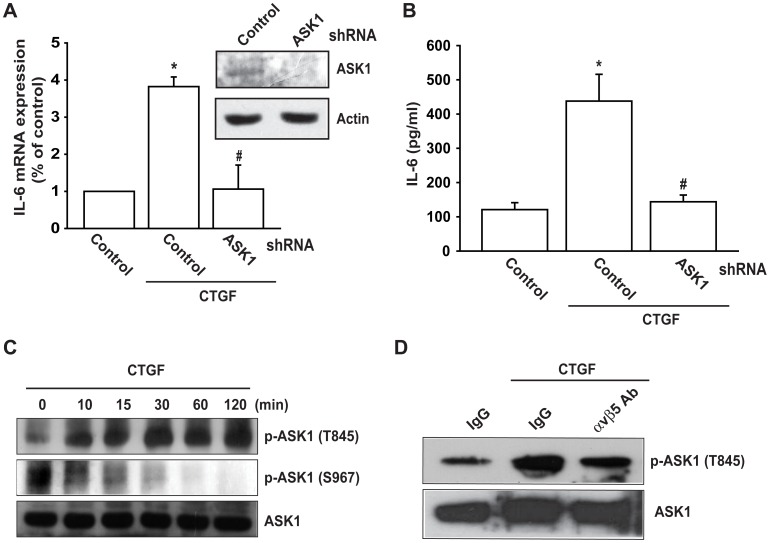
ASK1 is involved in CTGF-induced IL-6 production. (A&B) OASFs were transfected with ASK1 shRNA for 24 h, the IL-6 expression was examined by qPCR and ELISA. (C) OASFs were incubated with CTGF for indicated time intervals, and ASK1 phosphorylation was examined by Western blotting. (D) OASFs were pretreated with αvβ5 integrin antibody (5 µg/ml) for 30 min and then incubated with CTGF (10 ng/ml) for 30 min, and ASK1 phosphorylation was examined by Western blotting. *: p<0.05 as compared with basal level. #: p<0.05 as compared with CTGF-treated group.

Previous studies have shown that ASK1 plays a crucial role in regulating the expression of genes [27], [28]. To determine whether ASK1 is involved in CTGF-triggered IL-6 production, the ASK1 shRNA was used. Transfection of OASFs with ASK1 shRNA specifically blocked protein expression of ASK1 (Fig. 2A). In addition, ASK1 shRNA also reduced CTGF-induced IL-6 expression (Fig. 2A&B). We then directly measured phosphorylation of ASK1 in response to CTGF. ASK1 activation, as indicated by phosphorylation at the activation loop Thr^845^ and dephosphorylation at Ser^967^, was assessed by immunoblot analysis. Treatment of OASFs with CTGF significantly increased phosphorylation at Thr^845^ concomitant reduction of phosphorylation at Ser^967^ (Fig. 2C). Pretreatment of cells with αvβ5 mAb reduced CTGF-induced ASK1 phosphorylation of Thr^845^ (Fig. 2D). Based on these results, CTGF appears to act through αvβ5 integrin- and ASK1-dependent signaling pathway to enhance IL-6 production in human synovial fibroblasts.

### The JNK and p38 Signaling Pathways are Involved in the Potentiating Action of CTGF

**Figure 3:**
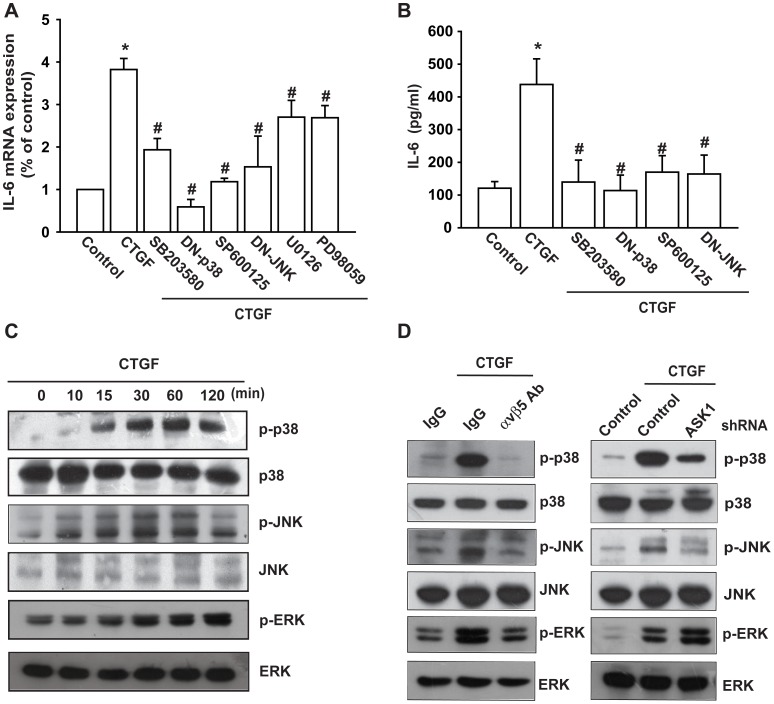
JNK and p38 are involved in CTGF-mediated IL-6 production in synovial fibroblasts. (A) OASFs were pretreated with SB203580, SP600125, U0126, and PD98059 for 30 min or transfected with p38 and JNK mutant for 24 h followed by stimulation with CTGF for 24 h. The IL-6 expression was examined by qPCR. (B) OASFs were pretreated with SB203580 and SP600125, for 30 min or transfected with p38 and JNK mutant for 24 h followed by stimulation with CTGF for 24 h. The IL-6 expression was examined by ELISA. (C) OASFs were incubated with CTGF for indicated time intervals, and JNK, p38, and ERK phosphorylation was examined by Western blotting. (D) OASFs were pretreated with αvβ5 integrin antibody (5 µg/ml) for 30 min or transfected with ASK1 shRNA for 24 h and then incubated with CTGF (10 ng/ml) for 30 min, and JNK, p38, and ERK phosphorylation was examined by Western blotting. *: p<0.05 as compared with basal level. #: p<0.05 as compared with CTGF-treated group.

ASK1 belongs to the MAPKKK family and activates the p38 and JNK pathways via MKK3/6 and MKK4/7, respectively [29]. We thus investigated the role of JNK and p38 in mediating CTGF-induced IL-6 expression using the specific JNK inhibitor SP600125 and p38 inhibitor SB203580. As shown in Figure 3A&B, CTGF-induced IL-6 expression was markedly attenuated by pretreatment of cells for 30 min with SP600125 and SB203580 or transfection of cells for 24 h with JNK and p38 mutant. Furthermore, MEK/ERK inhibitors U0126 and PD98059 also reduced CTGF-increased IL-6 expression (Fig. 3A). Stimulation of OASFs with CTGF resulted in a time-dependent phosphorylation of JNK, p38, and ERK (Fig. 3C). We next evaluated the relationship among αvβ5 integrin, ASK1, and JNK/p38/ERK in the CTGF-mediated signaling pathway and found that pretreatment of cells with αvβ5 mAb inhibited the CTGF-induced JNK, p38, and ERK phosphorylation (Fig. 3D). However, transfection of cells with ASK1 shRNA reduced the CTGF-induced JNK and p38 but not ERK phosphorylation (Fig. 3D). Therefore, JNK and p38 but not ERK are downstream molecules of CTGF-induced ASK1activation. Based on these results, CTGF appears to act via αvβ5 integrin receptor and the ASK1- and JNK/p38-dependent signaling pathway to enhance IL-6 production in human synovial fibroblasts.

### Involvement of NF-κB and AP-1 in CTGF-induced IL-6 Production

**Figure 4:**
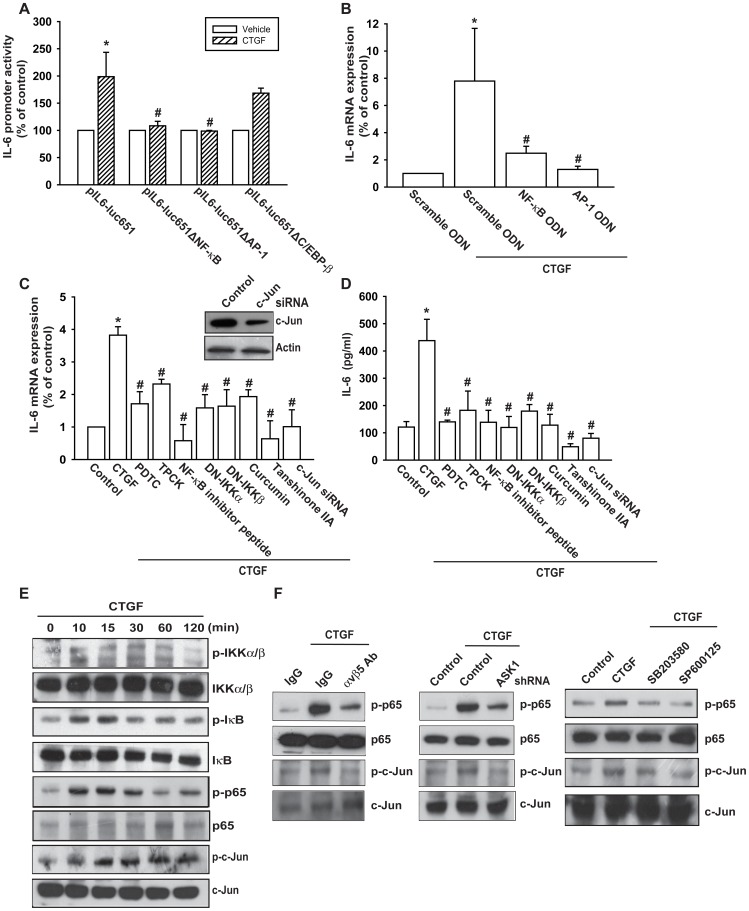
AP-1 and NF-κB are involved in the potentiation of IL-6 production by CTGF. (A) OASFs were transfected with IL-6 luciferase plasmids before incubation with CTGF for 24 h. Luciferase activity was then assayed. (B) OASFs were transfected with AP-1 or NF-κB ODN for 24 h and then incubated with CTGF (10 ng/ml) for 24 h, and IL-6 expression was examined by qPCR. (C&D) OASFs were pretreated for 30 min with TPCK (10 µM), PDTC (10 µM), NF-κB inhibitor peptide (10 µg/ml), Curcumin (10 µM), and Tanshinone IIA (10 µM) or transfected for 24 h with IKKα and IKKβ mutant and c-Jun siRNA followed by stimulation with CTGF for 24 h. The IL-6 expression was examined by qPCR and ELISA. (E) OASFs were incubated with CTGF for indicated time intervals, and total protein extracts were collected, and IKKα/β, IκBα, p65, and c-Jun phosphorylation was examined by Western blotting. (F) OASFs were pretreated with αvβ5 integrin antibody, SB203580, and SP600125 for 30 min or transfected with ASK1 shRNA for 24 h and then incubated with CTGF for 30 min, and total protein extracts were collected. The p65 and c-Jun phosphorylation was examined by Western blotting. *: p<0.05 as compared with basal level. #: p<0.05 as compared with CTGF-treated group.

The promoter region of human IL-6 contains three known *cis*-regulatory elements including AP-1, C/EBP-β, and NF-κB binding sites [10], [11]. Three different IL-6 promoter constructs containing mutations at NF-κB, C/EBP-β, or AP-1 sites respectively were generated by site directed mutagenesis. We found that CTGF-stimulated luciferase activity was abolished by NF-κB or AP-1 binding site mutation, but not by C/EBP-β site mutation (Fig. 4A). Therefore, NF-κB and AP-1 sites are more important in CTGF-mediated IL-6 expression. The increase of IL-6 mRNA expression by CTGF was antagonized by NF-κB and AP-1 ODN (Fig. 4B). Therefore, the NF-κB and AP-1 binding site are plays important role in CTGF-induced IL-6 production. The role of NF-κB and AP-1 were further established using the NF-κB inhibitor (PDTC), IκB protease inhibitor (TPCK), NF-κB inhibitor peptide, and AP-1 inhibitors (Curcumin [30] and Tanshinone IIA) or IKKα and IKKβ mutants or c-Jun siRNA and showed that these inhibitor, mutant, and siRNA blocked the enhancement of IL-6 production induced by CTGF (Fig. 4C&D). To rule out the off target effect of Curcumin and Tanshinone IIA on NF-κB and reactive oxygen species (ROS), we next examined the basal NF-κB and ROS activity after Curcumin and Tanshinone IIA treatment. However, Curcumin and Tanshinone IIA did not affect the basal level of NF-κB and ROS (the PDTC and NAC were used as positive control) (Fig. S2). On the other hand, stimulation of OASFs with CTGF increased IKKα/β, IκBα, p65, and c-Jun phosphorylation time-dependently (Fig. 4E). Pretreatment of cells with αvβ5 mAb, SB203580, and SP600125 or transfection of cells with ASK1 shRNA markedly inhibited the CTGF-induced p65 and c-Jun phosphorylation (Fig. 4F).

**Figure 5:**
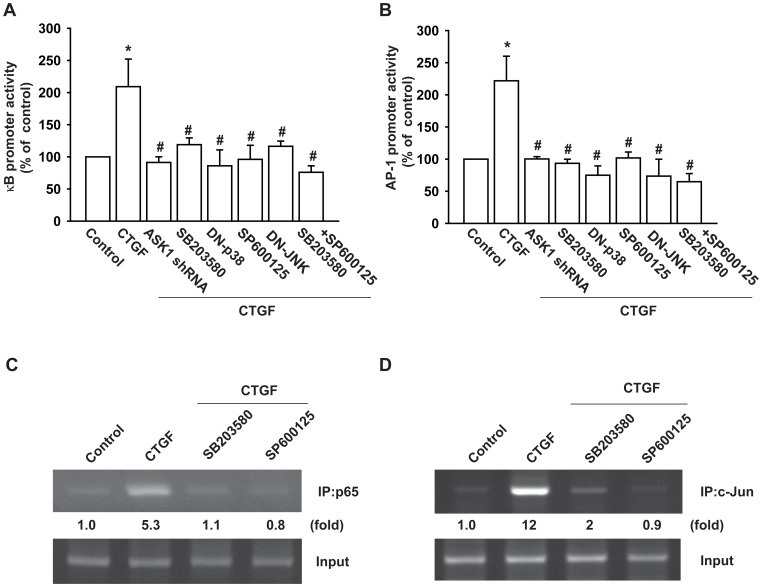
ASK1, p38, and JNK pathways are involved in CTGF-induced AP-1 and NF-κB activation. (A&B) OASFs were transfected with AP-1 and NF-κB-luciferase expression vector and then pretreated with SB203580, SP600125, SB203580 plus SP600125, or cotransfeced with JNK and p38 mutant or ASK1 shRNA before incubation with CTGF for 24 h. Luciferase activity was then assayed. (C&D) OASFs were pretreated with SB203580 and SP600125 then stimulated with CTGF for 120 min, and the chromatin immunoprecipitation assay was then performed. Chromatin was immunoprecipitated with anti-c-Jun or p65. One percentage of the precipitated chromatin was assayed to verify equal loading (input). *: p<0.05 as compared with basal level. #: p<0.05 as compared with CTGF-treated group.

NF-κB and AP-1 activation were further evaluated by analyzing the NF-κB and AP-1 luciferase activity, as well as by the chromatin immunoprecipitation assay. OASFs incubated with CTGF led to increase in NF-κB and AP-1 promoter activity (Fig. 5A&B). The increase of NF-κB and AP-1 activity by CTGF was reduced by ASK1 shRNA, SB203580, SP600125, p38 mutant, JNK mutant. In addition, combination of SB203580 and SP600125 complete reduced CTGF-induced NF-κB and AP-1 activity (Fig. 5A&B). Therefore, both p38 and JNK are mediated CTGF-induced NF-κB and AP-1 activation. We next investigated whether p65 and c-Jun binds to the NF-κB and AP-1 element on the IL-6 promoter after CTGF stimulation. The *in vivo* recruitment of p65 and c-Jun to the IL-6 promoter was assessed by the chromatin immunoprecipitation assay. *In vivo* binding of p65 to the NF-κB and c-Jun to the AP-1 element of the IL-6 promoter occurred after CTGF stimulation (Fig. 5C&D). The binding of p65 and c-Jun to the NF-κB (−288 to −39) and AP-1 (−312 to −39) elements by CTGF was attenuated by SB203580 and SP600125 (Fig. 5C&D). Taken together, these data suggest that that CTGF increased IL-6 production in OASFs via the αvβ5 integrin, ASK1, p38/JNK, and AP-1/NF-κB signaling pathways.

## Discussion

OA is a heterogeneous group of conditions associated with defective integrity of articular cartilage, in addition to related changes in the underlying bone. The chronic inflammatory process is mediated through a complex cytokine network. It is not yet completely understood that all the factors are responsible for initiating the degradation and loss of the articular tissues. CTGF has been involved in pathology of arthritis [15]. We confirmed that synovial fluid concentrations of CTGF were significantly higher in patients with OA than in normal fluid samples. We further identified IL-6 as a target protein for the CTGF signaling pathway that regulates the cell inflammatory response. We showed that potentiation of IL-6 by CTGF requires activation of the αvβ5 integrin, ASK1, p38/JNK, and AP-1/NF-κB signaling pathways. These findings suggest that CTGF acts as an inducer of inflammatory cytokines such as IL-6 and enhance the inflammatory response in OA.

Integrins, which link the extracellular matrix to intracellular signaling molecules, regulate a number of cellular processes, including adhesion, signaling, motility, survival, gene expression, growth, and differentiation. Integrins are known to serve as receptors for CTGF [31]. CTGF has been shown to bind integrin and increase gene expression [32]. Pretreatment of cells with anti-αvβ5 but not anti-αvβ3, α5β1, and α6β1 mAb inhibited the CTGF-induced IL-6 production. This indicates that integrin αvβ5 receptor played an important role in CTGF-induced IL-6 expression in OASFs.

ASK1 activity is regulated by multiple mechanisms including phosphorylation and interactions with various proteins. Phosphorylation at Ser^967^ is essential for ASK1 association with 14-3-3 protein, which attenuates ASK1 activity [33]. A previous study showed that ROS induces dephosphorylation of Ser^967^ as well as phosphorylation of Thr^845^ in the ASK1 activation loop, both of which are correlated with ASK1 activity [34]. Here, we found that CTGF enhanced dephosphorylation at Ser^967^ and phosphorylation at Thr^845^ of ASK1. Furthermore, ASK1 shRNA inhibited CTGF induced IL-6 expression. Therefore, ASK1 activation is required for CTGF induced IL-6 expression in human OASFs. ASK1 is an upstream molecule of JNK and p38, which have been shown to be involved in the regulation of genes expression [35]. We showed in present study that CTGF increased the p38 and JNK phosphorylation. Pretreatment of cells with p38 and JNK inhibitor antagonized the CTGF-induced IL-6 expression. This was further confirmed by the result that p38 and JNK mutant inhibited the enhancement of IL-6 by CTGF. On the other hand, αvβ5 mAb and ASK1 shRNA reduced CTGF-mediated JNK and p38 phosphorylation. Taken together, our results provide evidence that CTGF up-regulates IL-6 in human synovial fibroblasts via the αvβ5 integrin, ASK1, and JNK/p38 signaling pathway.

There are several binding sites for a number of transcription factors including NF-κB, CREB, NF-IL-6, and AP-1 box in the 5′ region of the IL-6 gene [10], [11]. Recent studies on the IL-6 promoter have demonstrated that IL-6 induction by several transcription factors occurs in a highly stimulus-specific or cell-specific manner [36]. The results of this study show that AP-1 and NF-κB activation contributes to CTGF-induced IL-6 production in synovial fibroblasts, and deletion of AP-1 and NF-κB site reduced CTGF-mediated IL-6 promoter activity. Pretreatment of cells with NF-κB inhibitors (PDTC and TPCK) or AP-1 inhibitors (Curcumin and Tanshinone IIA) also reduced CTGF-increased IL-6 production. Therefore, the AP-1 and NF-κB binding site are important in CTGF-induced IL-6 production. Furthermore, CTGF increased the binding of p65 to the NF-κB element and c-Jun to the AP-1 element on the IL-6 promoter, as shown by chromatin immunoprecipitation assay. Using transient transfection with AP-1 and NF-κB-luciferase as an indicator of AP-1 and NF-κB activity, we also found that CTGF induced an increase in AP-1 and NF-κB activity. In addition, ASK1 shRNA, SB203580, SP600125, p38 mutant, or JNK mutant reduced CTGF-increased AP-1 and NF-κB promoter activity. These results indicate that CTGF might act through the ASK1, p38/JNK, and AP-1/NF-κB pathway to induce IL-6 activation in human OASFs.

**Figure 6:**
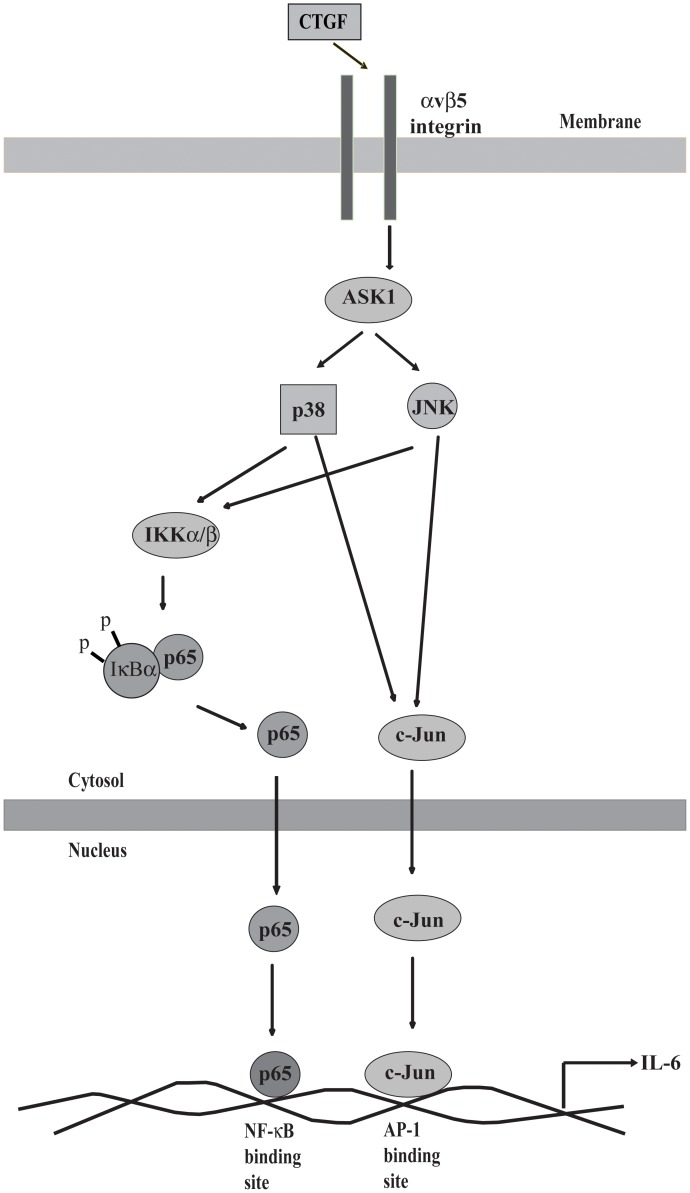
Schematic presentation of the signaling pathways involved in CTGF-induced IL-6 expression of synovial fibroblasts. CTGF and αvβ5 integrin interaction activates ASK1, and p38/JNK pathways, which enhances binding of p65 to the NF-κB site and c-Jun to the AP-1 site, resulting in the transactivation of IL-6 expression.

The development and progression of OA are now believed to involve inflammation even in the early stages of the disease [37]. Epidemiological studies show a clear link between the progression of tibiofemoral cartilage damage and the presence of a reactive or inflammatory synovium [38], [39]. Secreted inflammatory factors such as proinflammatory cytokines, therefore, are critical mediators of the disturbed metabolism and enhanced catabolism of joint tissue involved in OA. In this study, we have demonstrated for the first time that CTGF mediator the OASFs inflammatory by induce proinflammatory cytokine (IL-6). These results also indicate that CTGF might act through the αvβ5 integrin, ASK1, p38/JNK, and AP-1/NF-κB pathway to induce IL-6 activation in human OASFs (Fig. 6).

## Supporting Information

Figure S1 **The basal IL-6 expression after antibodies or inhibitors treatment.** OASFs were pretreated with IgG, αvβ3, αvβ5, α5β1, and α6β1 integrin antibody or vehicle (1% DMSO), SB203580, SP600125, U0126, PD98059, TPCK, PDTC, NF-κB inhibitor peptide, Curcumin, and Tanshinone IIA for 24 h. The IL-6 expression was examined by ELISA. Results are expressed as the mean ± S.E.
(TIF)

Figure S2 **The Curcumin and Tanshione IIA did not affect the basal level of NF-κB and ROS.** (A) OASFs were transfected with NF-κB-luciferase expression vector and then treated with Curcumin (10 µM), Tanshinone IIA (10 µM), or TPCK (10 µM) for 24 h. Luciferase activity was then assayed. (B) OASFs were pretreated with Curcumin (10 µM), Tanshinone IIA (10 µM), or NAC (10 mM) for 24 h and then labeled with DCF-DA (10 µM). The fluorescence intensity was measured by flow cytometry. *: p<0.05 as compared with basal level.
(TIF)
