# Correction: CTGF Increases IL-6 Expression in Human Synovial Fibroblasts through Integrin-Dependent Signaling Pathway

**DOI:** 10.1371/journal.pone.0144569

**Published:** 2015-12-11

**Authors:** Shan-Chi Liu, Chin-Jung Hsu, Hsien-Te Chen, Hsi-Kai Tsou, Show-Mei Chuang, Chih-Hsin Tang

The authors would like to correct Fig 3D, as errors were introduced in the preparation of this figure for publication. In Fig 3D, the left JNK panel appears as a duplicate of the right JNK panel. The authors have provided a corrected version of Fig 3 here.

The authors confirm that these changes do not alter their findings. The authors have provided raw, uncropped blots as Supporting Information.

**Fig 3:**
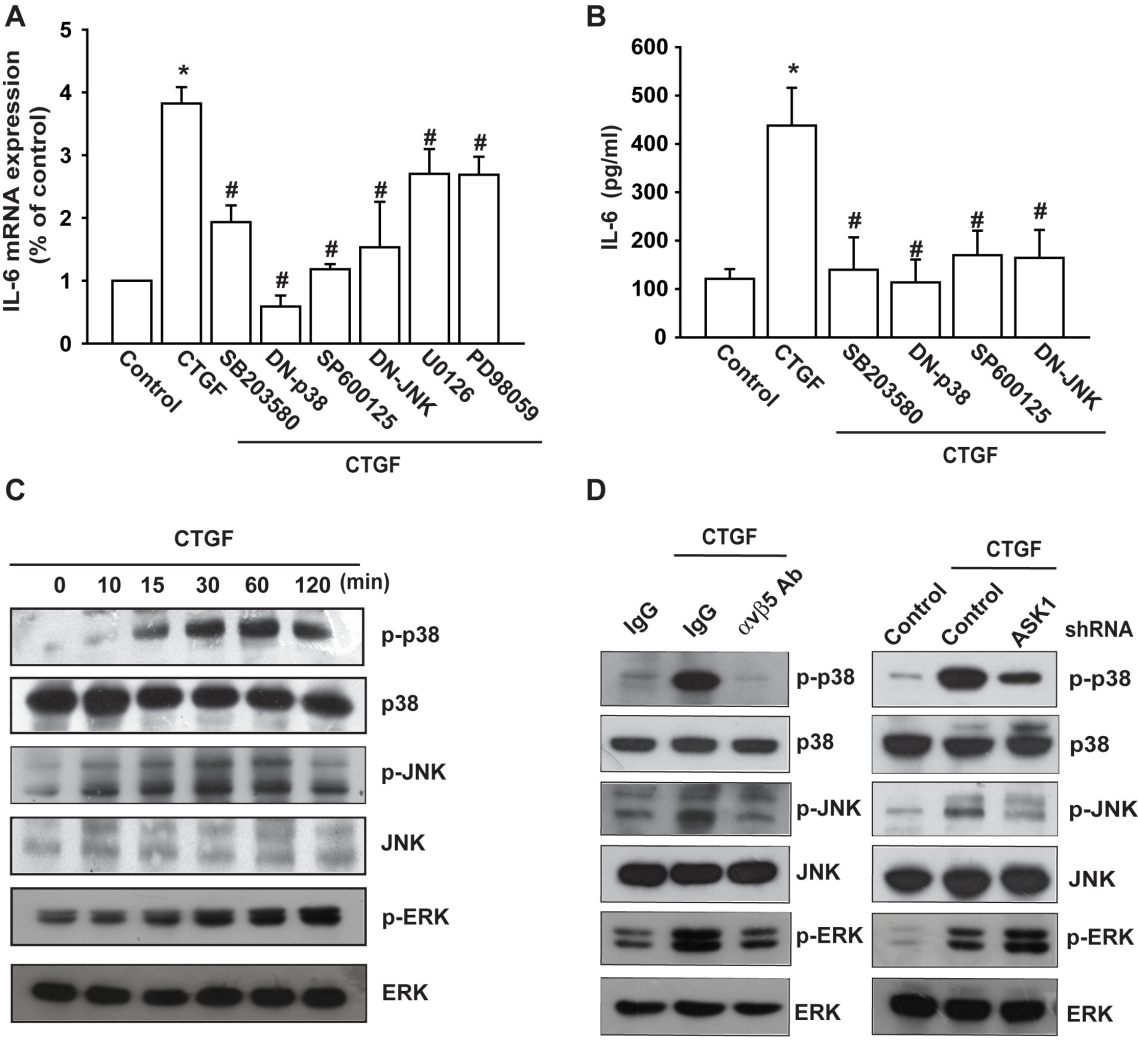
JNK and p38 are involved in CTGF-mediated IL-6 production in synovial fibroblasts. (A) OASFs were pretreated with SB203580, SP600125, U0126, and PD98059 for 30 min or transfected with p38 and JNK mutant for 24 h followed by stimulation with CTGF for 24 h. The IL-6 expression was examined by qPCR. (B) OASFs were pretreated with SB203580 and SP600125, for 30 min or transfected with p38 and JNK mutant for 24 h followed by stimulation with CTGF for 24 h. The IL-6 expression was examined by ELISA. (C) OASFs were incubated with CTGF for indicated time intervals, and JNK, p38, and ERK phosphorylation was examined by Western blotting. (D) OASFs were pretreated with αvβ5 integrin antibody (5 µg/ml) for 30 min or transfected with ASK1 shRNA for 24 h and then incubated with CTGF (10 ng/ml) for 30 min, and JNK, p38, and ERK phosphorylation was examined by Western blotting. *: p<0.05 as compared with basal level. #: p<0.05 as compared with CTGF-treated group.

## Supporting Information

S1 Fig Uncropped blots for Fig 3D. (TIF)

## References

[1] Liu S-C, Hsu C-J, Chen H-T, Tsou H-K, Chuang S-M, Tang C-H (2012) CTGF Increases IL-6 Expression in Human Synovial Fibroblasts through Integrin-Dependent Signaling Pathway. PLoS ONE 7(12): e51097 doi:10.1371/journal.pone.0051097 23227240 10.1371/journal.pone.0051097 PMC3515445

