# Supplementary figures and images for: CTGF Increases IL-6 Expression in Human Synovial Fibroblasts through Integrin-Dependent Signaling Pathway
Source: PLoS One. 2012 Dec 5;7(12):e51097. doi: 10.1371/journal.pone.0051097 (PMC3515445; doi:10.1371/journal.pone.0051097)

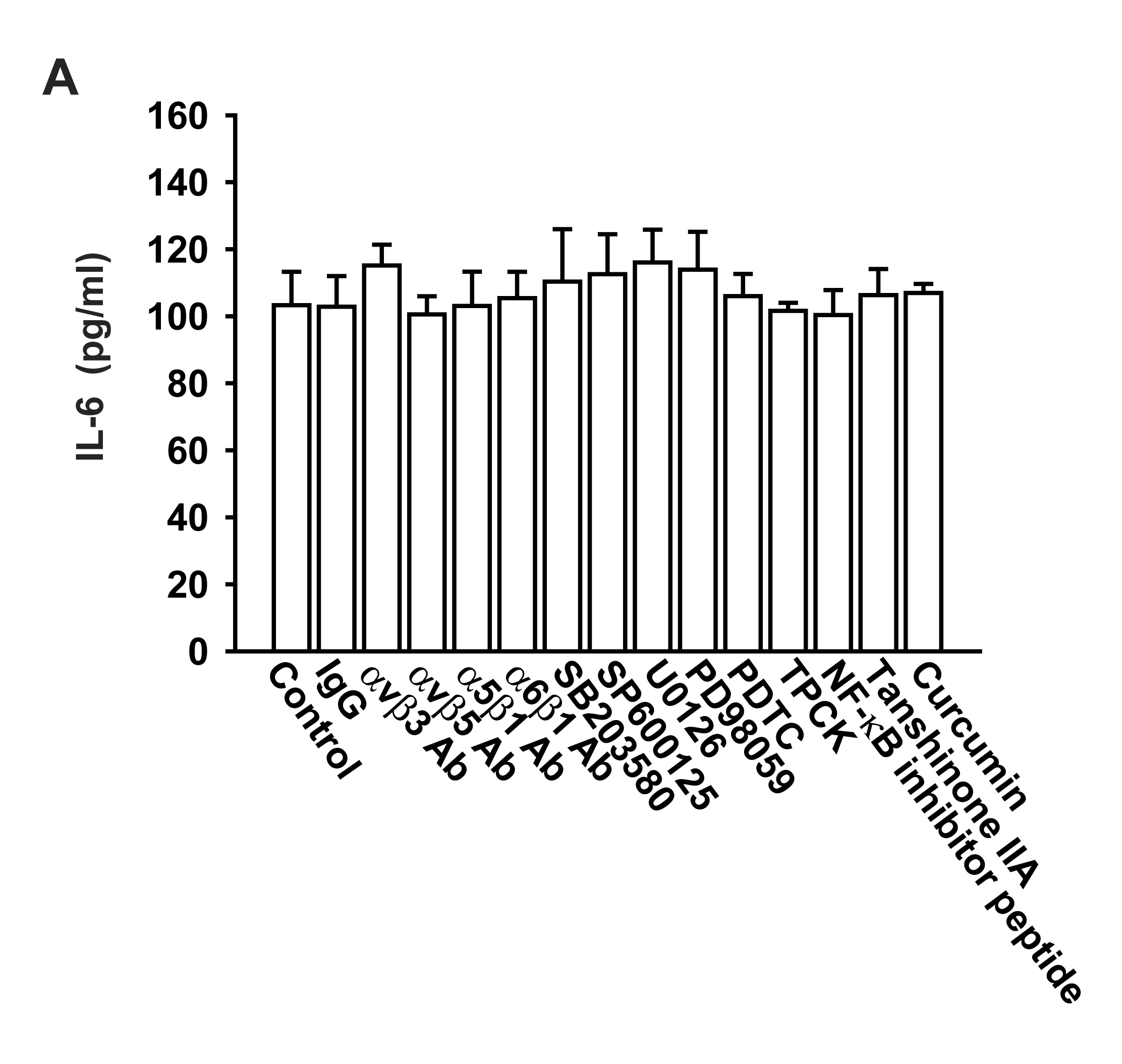

Supplement: Figure S1 — The basal IL-6 expression after antibodies or inhibitors treatment. OASFs were pretreated with IgG, αvβ3, αvβ5, α5β1, and α6β1 integrin antibody or vehicle (1% DMSO), SB203580, SP600125, U0126, PD98059, TPCK, PDTC, NF-κB inhibitor peptide, Curcumin, and Tanshinone IIA for 24 h. The IL-6 expression was examined by ELISA. Results are expressed as the mean ± S.E. (TIF) [file pone.0051097.s001.tif]

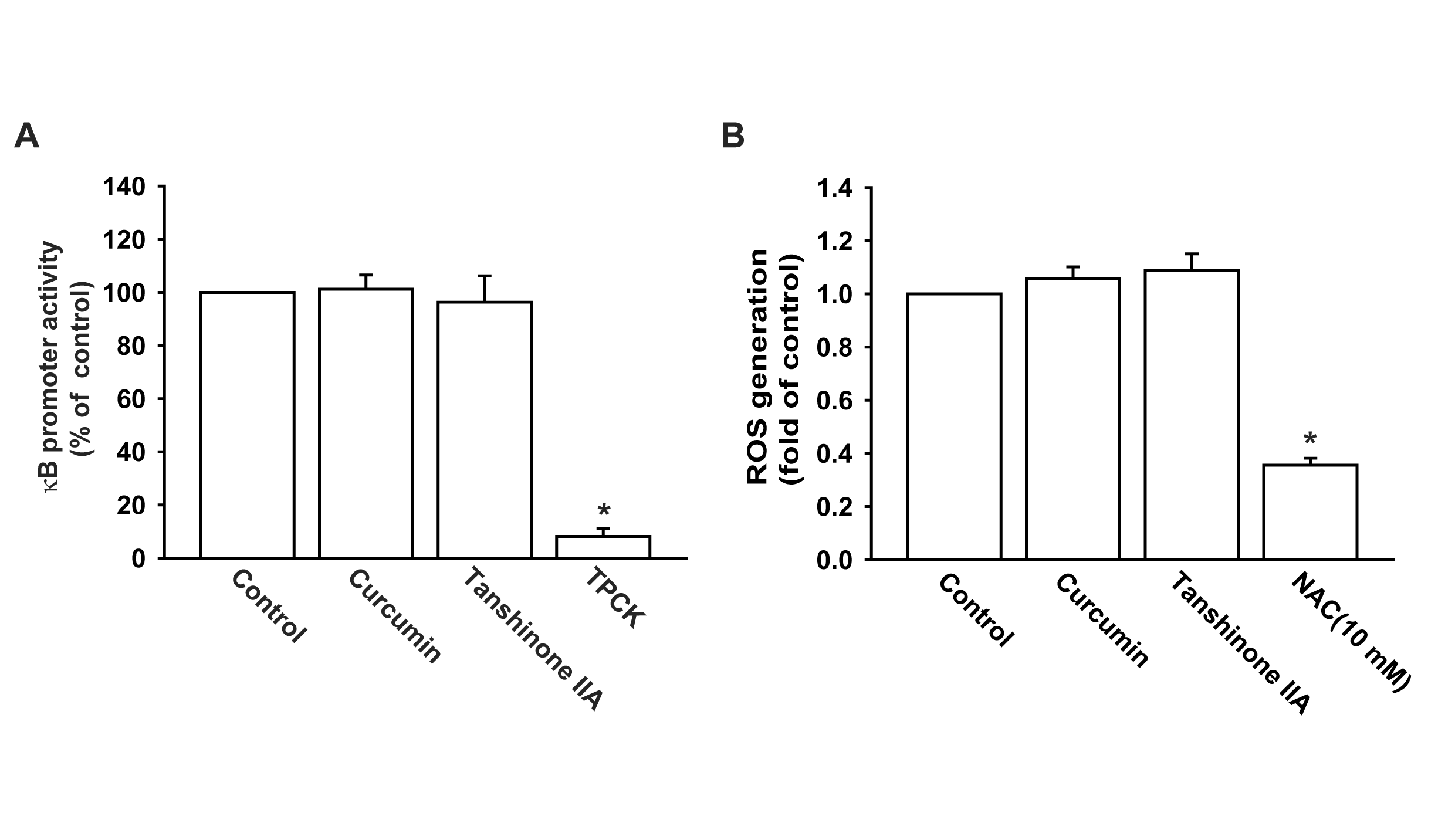

Supplement: Figure S2 — The Curcumin and Tanshione IIA did not affect the basal level of NF-κB and ROS. (A) OASFs were transfected with NF-κB-luciferase expression vector and then treated with Curcumin (10 µM), Tanshinone IIA (10 µM), or TPCK (10 µM) for 24 h. Luciferase activity was then assayed. (B) OASFs were pretreated with Curcumin (10 µM), Tanshinone IIA (10 µM), or NAC (10 mM) for 24 h and then labeled with DCF-DA (10 µM). The fluorescence intensity was measured by flow cytometry. *: p<0.05 as compared with basal level. (TIF) [file pone.0051097.s002.tif]
