# Supplementary figures and images for: Correction: CTGF Increases IL-6 Expression in Human Synovial Fibroblasts through Integrin-Dependent Signaling Pathway
Source: PLoS One. 2015 Dec 11;10(12):e0144569. doi: 10.1371/journal.pone.0144569 (PMC4686119; doi:10.1371/journal.pone.0144569)

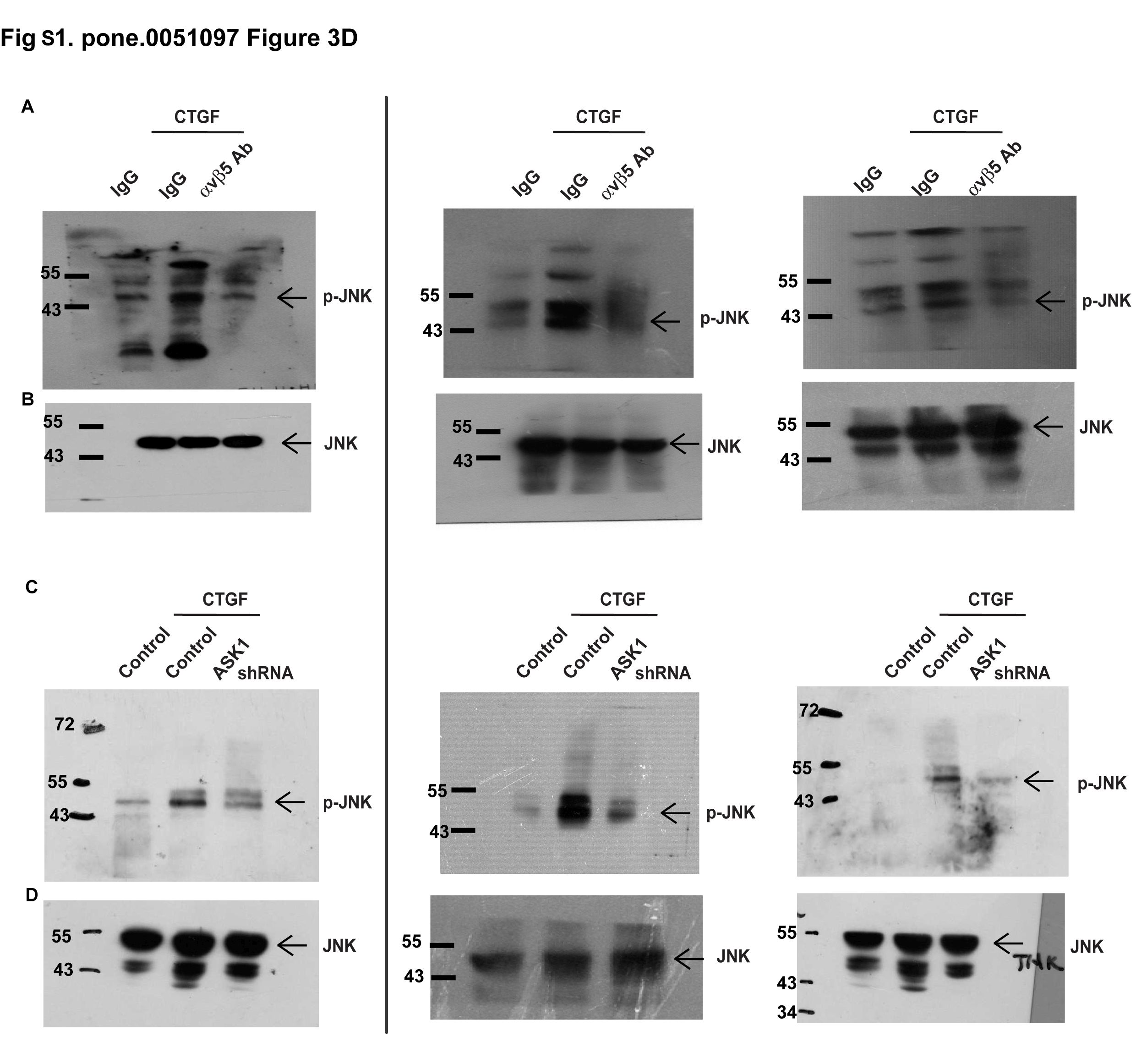

Supplement: S1 Fig — (TIF) [file pone.0144569.s001.tif]
